# Effect of dietary selenium intake on CVD: a retrospective cohort study based on China Health and Nutrition Survey (CHNS) data

**DOI:** 10.1017/S1368980024000703

**Published:** 2024-03-27

**Authors:** Yaqi Wen, Laixi Zhang, Shengping Li, Tiankun Wang, Ke Jiang, Lingxi Zhao, Yuzhao Zhu, Wen Zhao, Xun Lei, Manoj Sharma, Yong Zhao, Zumin Shi, Jun Yuan

**Affiliations:** 1 School of Public Health, Chongqing Medical University, Chongqing, China; 2 Research Center for Medicine and Social Development, Chongqing Medical University, Chongqing, China; 3 The Innovation Center for Social Risk Governance in Health, Chongqing Medical University, Chongqing, China; 4 Chongqing Key Laboratory of Child Nutrition and Health, Children’s Hospital of Chongqing Medical University, Chongqing, China; 5 Social & Behavioral Health, School of Public Health, University of Nevada, Las Vegas, NV, USA; 6 Human Nutrition Department, College of Health Sciences, QU Health, Qatar University, Doha, Qatar

**Keywords:** Dietary selenium intake, CVD, Impact, China

## Abstract

**Objective:**

We aimed to examine the association between dietary Se intake and CVD risk in Chinese adults.

**Design:**

This prospective cohort study included adults above 20 years old in the China Health and Nutrition Survey (CHNS), and they were followed up from 1997 to 2015 (*n* 16 030). Dietary data were retrieved from CHNS, and a 3-d, 24-h recall of food intake was used to assess the cumulative average intake of dietary Se, which was divided into quartiles. The Cox proportional hazards model was adopted to analyse the association between dietary Se intake and incident CVD risk.

**Setting:**

CHNS (1991, 1993, 1997, 2000, 2004, 2006, 2009, 2011 and 2015)

**Results:**

A total of 663 respondents developed CVD after being followed up for a mean of 9·9 years (median 9 years). The incidence of CVD was 4·3, 3·7, 4·6 and 4·0 per 1000 person-years across the quartiles of cumulative Se intake. After adjusting all potential factors, no significant associations were found between cumulative Se intake and CVD risk. No interactions were found between Se intake and income, urbanisation, sex, region, weight, hypertension and CVD risk.

**Conclusion:**

We found no association between dietary Se and CVD.

CVD is the leading cause of death worldwide, and the number of CVD deaths rose steadily from 12·1 million in 1990 to 18·6 million in 2019^(1)^. The incidence and mortality rates of CVD in China were at the top of the list, according to the 2021 annual report on cardiovascular health and disease, with rural and urban CVD accounting for 46·74 % and 44·26 % of deaths in 2019, respectively; two out of every five deaths were due to CVD, and the projected number of current patients with CVD is 0·33 billion^(2)^. More than 40 % of deaths were attributed to CVD between 1990 and 2016^(3)^. CVD is the major consequence of dietary risks, causing 7·94 million annual deaths in 2019^(4)^. Diet is a major influencing factor in CVD that is closely related to its occurrence^(5–7)^.

Se is a crucial component of selenoproteins with selenocysteine action and is involved in vital enzymatic processes like redox homoeostasis^(8)^. Many studies showed that Se is an important trace element that affects cardiovascular health because of the potential of selenoproteins, such as glutathione peroxidase and selenoprotein S, to protect against oxidative stress^(9)^. Indigenous people living in Canada and the USA are exposed to high Se levels through their traditional diet, which is rich in marine mammals and fish; blood and dietary Se are reversely associated with the prevalence of stroke amongst Inuits^(10)^. Se may exhibit a protective effect against mercury (Hg) in CVD^(11)^. However, other studies showed that chronic overexposure to environmental Se may increase the risk of elevated blood pressure and diabetes^(12,13)^. Moreover, globally, one in seven people has low-dietary Se intake^(14)^. Sixteen regions of China are included in the Se-deficient zone from the northeast to the southwest^(15)^. Se deficiency has long been associated with CVD. Cardiomyopathy, also known as Keshan disease, is prevalent in parts of China where the soil has insufficient levels of Se; in these regions, Se supplementation interventions have achieved prevention and treatment effects^(16)^.

However, experimental and observational studies have provided conflicting evidence on the associations of Se with the incidence and mortality of CVD. Some studies indicated that the increased CVD risk associated with low Se intake can be demonstrated by evaluating glutathione peroxidase activity in blood^(17)^. High Se levels in the body are associated with decreased risk for CVD incidence and mortality^(18)^. In addition, a meta-analysis in 2013 found that an adequate dietary intake of Se is essential to maintain a healthy ageing population, especially in terms of their cardiovascular health^(19,20)^. Other studies have found that dietary intake of Se may be negatively associated or not associated with cardiovascular mortality. High Se is associated with an increased risk of diabetes^(20,21)^ and hypertension and may induce toxicity^(22)^. In addition, some studies found no statistically significant effects of Se supplementation on CVD mortality, non-fatal CVD events or all CVD events (fatal and non-fatal)^(23)^. A review published in 2021 showed that data from clinical studies do not adequately demonstrate the beneficial effects of Se supplementation in the treatment or prevention of CVD^(9)^. Some studies validated that Se deficiency is associated with CVD^(24,25)^. However, other studies have reported a negative correlation or no relationship^(24,26,27)^ between Se and CVD.

In most population studies, the Se status is based on biomarkers, such as plasma Se concentrations, rather than dietary Se. Furthermore, the main source of Se in the human body is the diet, and about 80 % of Se in the diet is absorbed^(28)^. The beneficial effects of Se supplements are uncertain, and their arbitrary use has the potential for toxicity; further evidence is urgently required before prescribing Se supplements for cardiovascular health. We aimed to assess the association between Se intake and CVD prevalence in the Chinese adult population based on the China Health and Nutrition Survey (CHNS).

## Materials and methods

### Study design and population

Dietary Se data for this study were obtained from CHNS. The Chinese Center for Disease Control and Prevention’s National Institute for Nutrition and Food Safety and the Carolina Population Center at the University of North Carolina collaborated on the CHNS through a long-term follow-up study. The multistage stratified cluster random sampling procedure sampled over 30 000 people from 7,200 households in fifteen regions. Ten rounds of surveys were conducted (in 1989, 1991, 1993, 1997, 2000, 2004, 2006, 2009, 2011 and 2015). Given the availability of dietary information, the analysis in this research included respondents from 1991 to 2011 to explore the association between Se intake and CVD. A total of 25 252 participants (over the age of 20 years) were involved in all eight polls from 1991 to 2011. The 2015 data were only used to assess the incident CVD based on dietary intake between 1991 and 2011. It is a common approach when analysing longitudinal panel data.

**Fig. 1:**
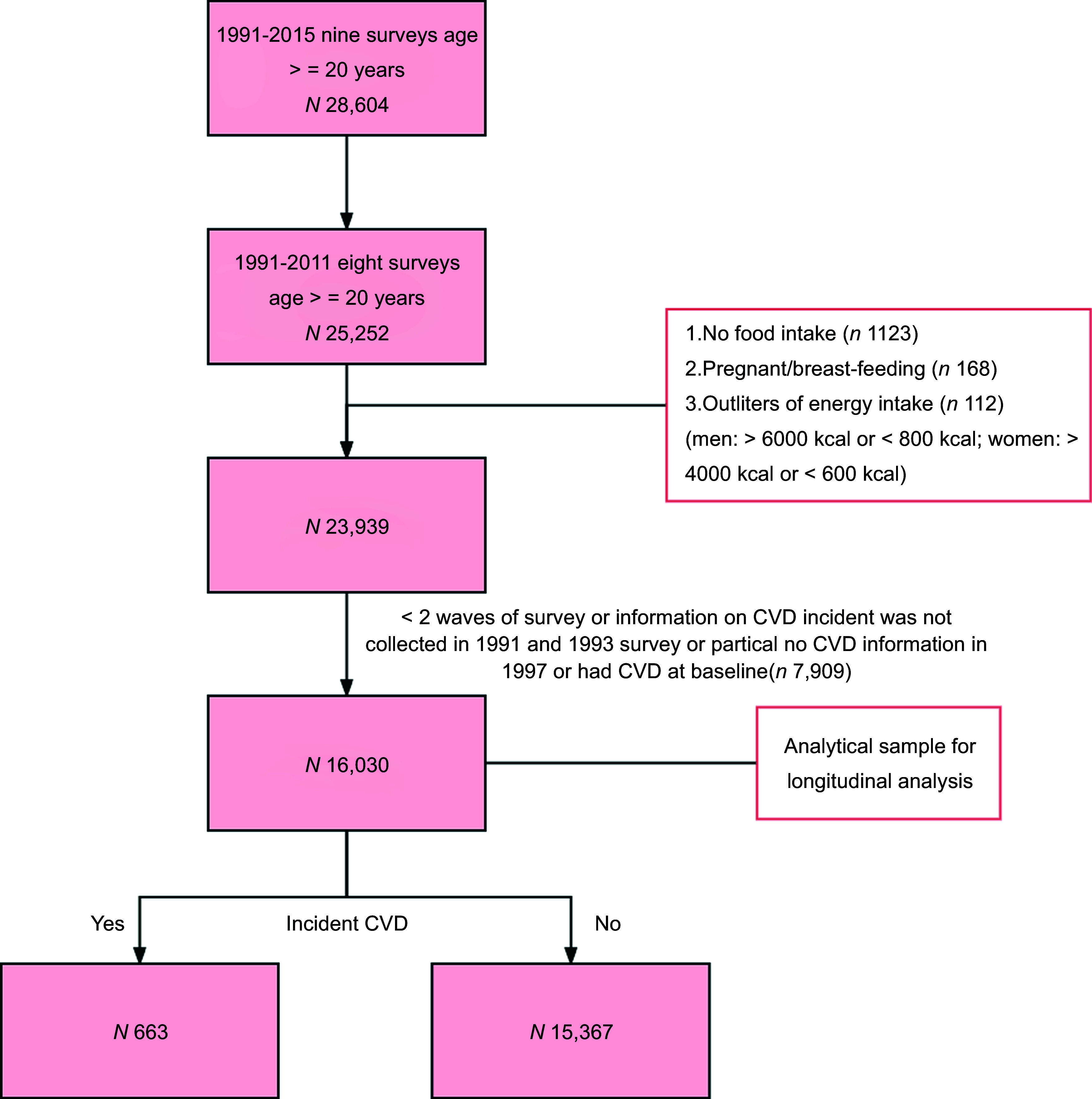
Participant flow chart

We excluded the subjects without food intake information (*n* 1,123), those with high calculations of their daily energy intake (men: <800 kcal or >6000 kcal; women: <600 or >4000 kcal) (*n* 112), pregnant/breast-feeding females (*n* 168) and those who participated in less than two waves of survey or information on CVD events was not collected in 1991 and 1993 or had partial no CVD information in 1997 or had CVD at baseline (*n* 7,909). This study used outcome data from the CHNS 1997–2015, and only participants who attended at least two rounds of survey between 1997 and 2015 were eligible for the analyses, because the CHNS is an open cohort so participants entered and left the cohort at any round of survey. Amongst the participants included in the analytical sample, 7,032 participants had food intake data between 1991 and 1993, as well as between 1997 and 2011. To reflect long-term dietary habits, we included the intake in 1991 and 1993 in the calculation of cumulative intake. A total of 16 030 samples were considered eligible for analysis (Fig. 1).

### Outcome variable: CVD

In this study, myocardial infarction and stroke were included in CVD by referring to previous studies on CVD using the CHNS database^(6)^. The diagnosis of CVD was based on self-reported history of stroke and/or myocardial infarction^(29)^. Self-reported history of CVD was collected by asking, ‘Has the doctor ever given you the diagnosis of myocardial infarction?’ or ‘Has the doctor ever given you the diagnosis of stroke?’.

### Exposure variables: dietary Se intake

Dietary Se intake was obtained from a 3-d, 24-h dietary survey and a household-based survey, thereby ensuring the accuracy of the dietary data source. For three consecutive days, each household member reported all the food they had eaten in the previous 24 h, both at home and outside. Meanwhile, a household’s food inventory was recorded by an investigator, including all available food stored, purchased and leftover food waste. Trained investigators used standard forms to collect details of intake (quantity and type of food, type and place of the meal). Investigators coded the information according to the Chinese Food Composition Table (i.e. FCT 1981, 1991, 2002 and 2004) to calculate cumulative Se intake as the exposure variable given the differences in nutrients in foods grown in various regions. We used cumulative Se intake as the exposure variable, which could effectively reduce the differences between individuals and represent the dietary habits of the subjects over time. For example, a person’s intake of Se was x1, x2 and x3 in 1997, 2000 and 2004, respectively. x1 represents the baseline intake; the cumulative average Se intake is x1 in 1997, (x1 + x2)/2 in 2000 and (x1 + x2 + x3)/3 in 2004; x1, x2 and x3 represent recent Se intake in 1997, 2000 and 2014, respectively^(30)^. The dietary intake data before 1997 were used to capture long-term intake via the cumulative average intake approach. This method was also used in previous studies^(31)^.

### Covariates

A structural questionnaire was used to collect covariates such as sociodemographics, dietary patterns, health status and lifestyle factors (China’s economy, population, nutrition and health survey – community survey). The socio-economic variables included per capita annual family income (low, medium and high); education was divided into high (high middle school and above), medium (junior middle school) and low (illiterate/primary school) levels; urbanisation was categorised into three levels (low, medium and high)^(32)^. Physical activity levels were measured by the metabolic equivalent of task, which was estimated based on self-reported activities (including transportation, occupation, family and recreation)^(33)^. The smoking condition was classified as non-smoker, ex-smoker and current smoker. The alcohol consumption choices were defined as abstaining, consuming one to two times per week, consuming three to four times per week and drinking all the days of the week. Obesity level was assessed by BMI, and overweight was defined as BMI > 24 kg/m^2^ (Chinese standards)^(34)^. Furthermore, the prevalence of diabetes or hypertension was calculated in each group. The south geographical regions included Jiangsu, Hubei, Hunan, Guizhou and Guangxi, whereas the north included Heilongjiang, Liaoning, Henan and Shandong^(35)^.

### Statistical analyses

Even though data on CVD incidents were not gathered in the surveys conducted in 1991 and 1993, dietary intake was considered in the calculation of cumulative intake to accurately reflect long-term dietary intake. We chose to include the dietary intake data from 1991 and 1993 in the analysis because, in sensitivity analyses, the main conclusions remained unchanged when the data from those years were excluded. Amongst the analytical sample, 7,032 participants had dietary data before 1997. The method has been used by previous studies^(36)^. The 2015 data were only used to assess the incidence of CVD based on dietary intake between 1991 and 2011. It is a common approach when analysing longitudinal panel data.

Stata software (version 17.0) was used to perform all statistical analyses. The dietary cumulative average intake of dietary Se was divided into quartiles. The mean and standard deviation was used to describe the continuous variables, and the categorical variables were determined in terms of frequency and proportion (%). The continuous variables were tested by ANOVA or Kruskal–Wallis test, and the proportions for the *χ* ^2^ test were employed to compare the categorical variables. The Cox proportional hazards model with time-varying cumulative Se intake and covariates was used to calculate the hazard ratio for incident CVD. Four models were built, as follows: model 1 was adjusted for age, sex and energy intake. Model 2 was further adjusted for intake of fat, smoking, alcohol drinking, income, urbanisation, education and physical activity. Model 3 was adjusted for intake of fruit and vegetables. Model 4 was further adjusted for BMI, diabetes and hypertension. The data on CVD were also obtained from CHNS, and the outcome variables of CVD were myocardial infarction and stroke. In the interactive analysis of Se intake and CVD risk, the Cox proportional hazards model was used to analyse the association between incident CVD risk and dietary Se intake. The model was adjusted for age, sex, energy intake, intake of fat, smoking, alcohol drinking, income, urbanisation, physical activity, intake of fruit and vegetables, BMI, diabetes and hypertension. Moreover, we added energy intake in the multivariable Cox regression model. We did not use the energy residual method to calculate the energy-adjusted intake. The related models were unadjusted for stratification variables. *P* < 0·05 (two-tailed) was significant for all analyses conducted.

## Results

Table 1 shows that different participants may have varying survey years as the baseline. The last year of the survey was 2015. At baseline, 16 030 participants free of CVD were included in the analyses. The incidence values of CVD were 4·3, 3·7, 4·6 and 4·0 per 1000 person-years across the quartiles of cumulative Se intake. Across quartiles of Se intake, the mean values (s d) of Se intake were 20·2 ± 5·0, 32·0 ± 2·9, 42·8 ± 3·6 and 72·0 ± 47·0. Table 1 shows that hypertension was not significantly different from cumulative Se intake, but other variables had a positive association with cumulative Se intake (*P* < 0·05). With increasing cumulative Se intake, the age decreased, and men had a higher Se intake than women. Individuals in the fourth quartile of Se intake were more likely (*P* < 0·001) to have a higher intake of energy, fruit, fresh vegetables, meat and macronutrients (proteins, carbohydrates and fats) and were younger (42·3 ± 14·1) than their counterparts. The prevalence rates of overweight (39·8 %), alcohol drinking (44·7 %) and current smoking (35·6 %) were also higher amongst those with high Se intake. In the highest quartile of cumulative Se intake, diabetes, income and BMI were higher than those in the lowest quartile. In addition, the participants from the south had lower Se intake than those from the north.

**Table 1:** Baseline sample characteristics of Chinese adults attending CHNS by quartiles of cumulative selenium intake (*n* 16 030)

Factors	Q1 (*n* 4,026)		Q2 (*n* 4,032)		Q3 (*n* 4,011)		Q4 (*n* 3,961)		*P*-value
	*n*	%	*n*	%	*n*	%	*n*	%	
Age (years)*	45·5	15·6	43·9	14·9	43·1	14·4	42·3	14·1	<0·001
Sex									<0·001
Men	1,530	38·0	1,772	43·9	2,114	52·7	2,370	59·8	
Women	2,496	62·0	2,260	56·1	1,897	47·3	1,591	40·2	
Income									<0·001
Low	1,549	38·9	1,149	28·8	934	23·5	764	19·5	
Medium	1,326	33·3	1,401	35·1	1,340	33·7	1,164	29·8	
High	1,103	27·7	1,444	36·2	1,708	42·9	1,982	50·7	
Education									<0·001
Low	2,034	54·8	1,530	41·6	1,298	34·9	1,017	27·5	
Medium	1,010	27·2	1,223	33·3	1,264	34·0	1,288	34·8	
High	665	17·9	923	25·1	1,154	31·1	1,398	37·8	
Urbanisation									<0·001
Low	1,731	43·0	1,212	30·1	950	23·7	753	19·0	
Medium	1,097	27·2	1,206	29·9	1,095	27·3	963	24·3	
High	1,198	29·8	1,614	40·0	1,966	49·0	2,245	56·7	
Smoking									<0·001
Non-smoker	2,918	72·7	2,810	69·8	2,620	65·5	2,459	62·1	
Ex-smokers	80	2·0	70	1·7	73	1·8	90	2·3	
Current smokers	1,018	25·3	1,143	28·4	1,310	32·7	1,408	35·6	
Survey year									<0·001
1997	2,008	49·9	1,924	47·7	1,855	46·2	1,462	36·9	
2000	544	13·5	597	14·8	528	13·2	524	13·2	
2004	377	9·4	350	8·7	357	8·9	499	12·6	
2006	151	3·8	167	4·1	207	5·2	253	6·4	
2009	239	5·9	310	7·7	372	9·3	453	11·4	
2011	707	17·6	684	17·0	692	17·3	770	19·4	
Alcohol drinking	1,089	27·5	1,311	33·0	1,524	38·6	1,741	44·7	<0·001
Region									<0·001
North	1,266	40·7	1,386	45·1	1,504	49·3	1,673	55·9	
South	1,848	59·3	1,685	54·9	1,544	50·7	1,320	44·1	
Physical activity (MET)*	145·6	118·6	139·7	115·2	129·3	107·3	129·3	109·3	<0·001
BMI (kg/m^2^)*	22·5	3·3	22·9	3·3	23·0	3·3	23·4	3·4	<0·001
Overweight	1,073	28·9	1,219	32·3	1,333	35·7	1,471	39·8	<0·001
Energy intake (kcal/d)*	1798·1	559·1	2068·3	565·8	2284·1	600·8	2548·6	687·3	<0·001
Fat intake (g/d)*	51·7	30·9	63·2	31·3	72·6	33·5	87·1	41·1	<0·001
Protein intake (g/d)*	48·3	14·6	61·9	15·0	72·7	16·9	89·4	24·2	<0·001
Carbohydrate intake (g/d)*	282·9	114·7	309·3	117·9	330·1	127·1	344·4	131·6	<0·001
Fe intake (g/d)*	15·5	6·4	19·2	7·4	21·9	8·9	27·5	19·0	<0·001
Intake of fruit (g/d)*	19·1	77·9	27·2	78·3	40·1	95·1	52·1	108·5	<0·001
Intake of fresh vegetable (g/d)*	258·4	159·7	264·7	169·9	280·4	170·5	303·5	179·2	<0·001
Intake of meat (g/d)*	39·2	46·6	72·3	64·5	99·0	79·4	143·8	121·1	<0·001
Se intake (μg/d) *	20·2	5·0	32·0	2·9	42·8	3·6	72·0	47·0	<0·001
Hypertension	610	16·3	635	16·7	646	17·2	690	18·5	0·059
Diabetes	73	1·8	70	1·8	81	2·1	110	2·8	0·004

CHNS, China Health and Nutrition Survey.

Data are presented as *mean (sd) for continuous measures and *n* (%) for categorical measures.

After follow-up for a mean of 9·9 years (median = 9 years), with 158 915 person-years follow-up, 663 incident CVD cases were recorded. Across the cumulative Se intake quartiles, CVD incidence was 4·3, 3·7, 4·6 and 4·0 per 1000 person-years. The cumulative Se intake had a statistically significant association with CVD after adjusting for age, sex and energy intake (*P* < 0·05). Compared with the first quartile of Se intake, the hazard ratios and 95 % CI for CVD were 0·93 (95 % CI: 0·75, 1·16) in the second quartile, 1·22 (95 % CI: 0·98, 1·52) in the third quartile and 1·18 (95 % CI: 0·94, 1·49) in the fourth quartile. After adjusting all potential factors, no significance was found between cumulative Se intake and CVD risk (Table 2). In the subgroup analyses, we found that the association between models (after adjusting for all the covariates) and CVD was consistent (Table 3). No interactions were found amongst Se intake and income, urbanisation, sex, region, overweight, hypertension, age and CVD risk.

**Table 2:** Hazard ratios (HR) (95 % CI) for CVD risk according to quartiles of selenium intake in China Nutrition and Health Study, 1997–2015

	Quartiles of Se intake							
		Q2		Q3		Q4		
	Q1	HR	95 % CI	HR	95 % CI	HR	95 % CI	*P*_for trend_
Cases	173	149		181		160		
Person-years	40 488	39 815		39 052		39 560		
Incidence (per 1000)	4·3	3·7		4·6		4·0		
Model 1	1·00	0·93	0·75, 1·16	1·22	0·98, 1·52	1·18	0·94, 1·49	0·040
Model 2	1·00	0·93	0·72, 1·20	1·27	0·98, 1·63	1·12	0·85, 1·48	0·133
Model 3	1·00	0·93	0·72, 1·21	1·28	0·99, 1·65	1·14	0·86, 1·50	0·107
Model 4	1·00	0·87	0·67, 1·13	1·12	0·86, 1·45	0·93	0·70, 1·23	0·928

Effect estimates were hazard ratios (95 % CI) derived from multivariable Cox regressions. Model 1 adjusted for age, sex and energy intake. Model 2 further adjusted for fat intake, smoking, alcohol drinking, income, urbanisation, education and physical activity. Model 3 further adjusted for intake of fruit and vegetable. Model 4 further adjusted for BMI, diabetes and hypertension.

**Table 3:** Association between selenium intake and CVD risk in China Nutrition and Health Study, 1997–2015

	Quartiles of Se intake								*P*_for interaction_
	Q1	Q2	95 % CI	Q3	95 % CI	Q4	95 % CI	*P*_for trend_	
Income									0·490
Low	1·00	0·98	0·64, 1·50	1·18	0·75, 1·85	0·85	0·50, 1·44	0·834	
Medium	1·00	0·81	0·52, 1·28	0·99	0·62, 1·56	1·13	0·70, 1·84	0·500	
High	1·00	0·71	0·44, 1·15	1·04	0·67, 1·61	0·76	0·47, 1·23	0·611	
Urbanisation									0·905
Low	1·00	1·01	0·64, 1·62	1·05	0·63, 1·74	0·89	0·49, 1·60	0·793	
Medium	1·00	0·80	0·51, 1·26	1·00	0·63, 1·58	0·62	0·36, 1·07	0·203	
High	1·00	0·82	0·54, 1·27	1·21	0·81, 1·83	1·14	0·74, 1·76	0·218	
Sex									0·967
Men	1·00	0·83	0·55, 1·24	1·06	0·72, 1·55	0·89	0·60, 1·34	0·927	
Women	1·00	0·88	0·62, 1·23	1·13	0·79, 1·62	0·93	0·61, 1·42	0·879	
Education									0·039
Low	1·00	1·02	0·76, 1·38	0·96	0·68, 1·34	1·09	0·76, 1·57	0·782	
Medium	1·00	0·58	0·32, 1·07	1·12	0·66, 1·91	0·57	0·31, 1·05	0·353	
High	1·00	0·51	0·21, 1·22	1·20	0·57, 2·50	0·87	0·41, 1·86	0·665	
Region									0·157
North	1·00	1·05	0·71, 1·55	1·08	0·73, 1·58	0·75	0·49, 1·14	0·173	
South	1·00	0·70	0·46, 1·05	0·91	0·59, 1·39	0·93	0·57, 1·51	0·959	
Overweight									0·721
No	1·00	0·88	0·61, 1·27	0·96	0·66, 1·40	0·86	0·56, 1·32	0·607	
Yes	1·00	0·81	0·56, 1·18	1·19	0·83, 1·69	0·95	0·65, 1·40	0·709	
Hypertension									0·497
No	1·00	0·74	0·51, 1·09	0·93	0·63, 1·35	0·84	0·55, 1·26	0·636	
Yes	1·00	0·98	0·69, 1·40	1·25	0·88, 1·79	0·97	0·65, 1·45	0·787	
Age group									0·574
<40	1·00	0·22	0·02, 2·02	1·26	0·32, 5·00	0·84	0·18, 3·93	0·722	
>=40	1·00	0·87	0·67, 1·13	1·07	0·83, 1·40	0·91	0·68, 1·22	0·926	

Models were adjusted in terms of age, sex and energy intake, intake of fat, smoking, alcohol drinking, income, urbanisation, education, physical activity, intake of fruit and vegetable, BMI, diabetes, and hypertension. Stratification variables were not adjusted in the corresponding models.

## Discussion

Given the inconsistent conclusions of the association between Se and CVD, this population-based large prospective cohort study with 16 030 Chinese adults examined the relationship between dietary Se and CVD. Our study showed no correlation between Se and CVD, but we also found some meaningful results. The dietary Se intake of the population included in this study was generally lower than the recommended dietary Se intake of 60 μg/d by the Chinese Nutrition Society. The cumulative average Se intake of the population in northern China was higher than that in southern China, which was consistent with the low level of Se content in soil in China and the differences in the local areas between north and south^(37)^. The elderly with lower income or living in a lower urbanisation had lower cumulative Se intake than their counterparts.

In line with previous research, our study found no association between dietary Se and CVD. A meta-analysis of trials revealed no association of Se alone with CVD and all-cause mortality. Interestingly, adding Se to the antioxidant mix is significant in reducing all-cause death from CVD^(38)^. By contrast, some researchers found that dietary Se intake is inversely associated with cardiovascular mortality, CHD risk, chronic heart failure, angina, heart attack and stroke^(39–41)^. Se constitutes a dietary factor with protective action against cardiovascular pathologies in several animals and epidemiological studies^(42)^. However, both factors were compared to determine whether the Se factor is superior to the medical and lifestyle factors in the rates of CVD; the results showed that medical and lifestyle factors are much stronger determinants than Se^(43)^.

Additional studies are required to explore the association between dietary Se and CVD. Differences in Se intake and status amongst different groups may be the cause of the inconsistent results regarding the relationship between Se levels and cardiovascular events in various studies^(44)^. Although complicated, relationships between Se status or intake and health or disease risk need to be clarified to guide clinical practice, improve dietary advice and create successful public health policies^(45)^. Randomised controlled trials that consider all of these elements are essential, which involve extended periods of follow-up, measurement of various cardiometabolic effects and a sample size that is substantial enough to ensure adequate statistical power^(46)^. Therefore, Se supplements are not advisable to prevent CVD, and their excessive consumption could lead to an increased likelihood of Se toxicity^(47)^.

Various potential variables can affect the lack of an association between dietary Se and CVD. According to a study, elevated levels of some intermediate CVD risk factors, such as dyslipidaemia and type 2 diabetes, may be linked to high Se concentrations, which can reduce the inverse association and even raise the risk of CVD^(44)^. Despite being potential confounders, altering BMI, smoking, alcohol consumption, diabetes and hypertension did not alter the strength of the associations. We assumed that certain diseases and intestinal micro-organisms reduced the association of dietary Se with CVD, explaining why no correlation was found between the two. A study on Se and stroke revealed that the inverse association between non-linear and anaemia might diminish the potential impact of Se intake on stroke^(48)^. This condition may be due to the erythrocytes in the process of transportation and utilisation of Se with closely related roles, that is, the shrinking of the erythrocyte causes anaemia. Furthermore, the physiological effects of Se are affected. However, in this study, patients with anaemia symptoms were still included in the population, thereby causing a lack of a significant correlation between the two.

Se deficiency is common amongst Chinese residents. Dietary Se intake should be increased to 50–60 μg/d in the general population of China to maximise stroke prevention and maintain health, and the association between Se and stroke was stronger in the group with high Se intake than in the group with low intake^(41)^. This result was inconsistent with the findings of our study, possibly because of the samples and research period between the two studies. Notably, the national working conference of ‘Prevention and Control of Diseases, Quantitative Se Supplementation’, initiated by the Chinese Academy of Agricultural Sciences and supported by China’s high-tech industrialisation and national torch programme, was held in the Great Hall of the People to officially launch the national Se supplementation project in 2005. With the strong support of governments and relevant departments at all levels and the joint efforts of Se supplementation offices across the country, Se supplementation offices for all were established in twenty-nine provinces, cities and autonomous regions in 2013^(49)^. Since then, the Chinese population’s intake of Se has increased greatly, with more people reaching 50–60 μg/d because of these national universal Se supplementation measures.

This study’s strengths should be emphasised. First, most existing studies focused on the association between nail or blood/serum Se and CVD, but our study added to the library of dietary Se and CVD studies. Second, although many studies have examined the present level of Se, our research focused on investigating the long-term implications. Third, we examined the interaction by examining the connection between CVD risk and Se intake in various subgroups.

This study’s shortcomings should also be considered. First, there may be recall bias in the information provided by study participants regarding their CVD history and other self-reported variables. Second, self-reported diagnosis of CVD could be non-fatal CVD. Further, this study did not take fatal CVD into account. Third, the main indicators of CVD selected in this study were stroke and myocardial infarction (two categories of CVD). The association between dietary Se and CVD may be negatively affected because we reduced the cardiovascular scope artificially. Fourth, bias was inevitable because stroke and myocardial infarction were self-reported. Finally, the lack of a region-specific food composition table was a limitation of the study. However, with the rapid development of the transportation system in China, the food market is not localised. The food produced in the south can be easily available in the north. In 2019, a survey found that residents of four survey villages in Arong Banner, a typical district within China’s low Se belt, purchased the vast majority of their daily food from external sources, and all of their staple food was purchased externally^(50)^. The use of a region-specific food composition table in a nationwide nutrition survey may be impossible. Future research should use biomarkers to validate our findings.

### Conclusion

This study with a large population-based sample of longitudinal analysis was a representative examination of the association between dietary Se and CVD amongst persons (age ≥ 20 years) from nine provinces of China. We found no association between dietary Se and CVD. Low Se intake in a low Se population does not lead to a significant reduction of risk.
